# Assessing micro- vs macro-costing approaches for treating appendicitis in children with appendicectomy or non-operatively

**DOI:** 10.1007/s11136-023-03442-w

**Published:** 2023-06-07

**Authors:** Maria Chorozoglou, Isabel Reading, Simon Eaton, Shehryer Naqvi, Caroline Pardy, Keren Sloan, Christina Major, Natasha Demellweek, Nigel J. Hall

**Affiliations:** 1 Faculty of Medicine, University of Southampton, Southampton, UK; 2 School of Primary Care, Population Sciences and Medical Education, Faculty of Medicine, University of Southampton, Southampton, UK; 3 Department of Population Health Sciences, University College London Great Ormond Street Institute of Child Health, University College London, London, UK; 4 St George’s University Hospital NHS Foundation Trust, London, UK; 5 Southampton Children’s Hospital, Southampton, UK; 6 Alder Hey Children’s Hospital, Liverpool, UK

**Keywords:** Costing methodology, Micro-costing, Patient-Level Information and Costing Systems (PLICS), NHS Reference costs, Hospital administrative data, HRQoL and QALY, B41, C18, H43, I10

## Abstract

**Objectives:**

We conducted a health economic sub-study within a feasibility RCT comparing a non-operative treatment pathway as an alternative to appendicectomy for the treatment of uncomplicated acute appendicitis in children. The objectives were to understand and assess data collection tools and methods and to determine indicative costs and benefits assessing the feasibility of conducting a full economic evaluation within the definitive trial.

**Methods:**

We compared different methods of estimating treatment costs including micro-costing, hospital administrative data (PLICS) and health system (NHS) reference costs. We compared two different HRQoL instruments (CHU-9D and EQ-5D-5L) in terms of data completeness and sensitivity to change over time, including potential ceiling effects. We also explored how the timing of data collection and duration of the analysis could affect QALYs (Quality Adjusted Life Years) and the results of the cost-utility analysis (CUA) within the future RCT.

**Results:**

Using a micro-costing approach, the total per treatment costs were in alignment with hospital administrative data (PLICS). Average health system reference cost data (macro-costing using NHS costs) could potentially underestimate these treatment costs, particularly for non-operative treatment. Costs incurred following hospital discharge in the primary care setting were minimal, and limited family borne costs were reported by parents/carers. While both HRQoL instruments performed relatively well, our results highlight the problem of ceiling effect and the importance of the timing of data collection and the duration of the analysis in any future assessment using QALYs and CUA.

**Conclusions:**

We highlighted the importance of obtaining accurate individual-patient cost data when conducting economic evaluations. Our results suggest that timing of data collection and duration of the assessment are important considerations when evaluating cost-effectiveness and reporting cost per QALY.

**Clinical trial registration:**

Current Controlled Trials ISRCTN15830435.

**Supplementary Information:**

The online version contains supplementary material available at 10.1007/s11136-023-03442-w.

## Plain English summary

Appendicitis is one of the most common acute surgical emergencies in children. Treatment traditionally includes appendicectomy. We undertook a feasibility study to explore a non-operative treatment pathway compared to appendicectomy. The feasibility economic sub-study describes the assessment of different data collection tools and methods to be used in a future clinical study to assess these different treatment options. The study highlighted the importance of individual-patient cost data for economic evaluations alongside clinical studies. The study also found that the timing of data collection and duration of the assessment were essential for assessing and reporting quality of life in this context. In addition, the importance of research nurse support in collecting this data from participants was highlighted.

## Introduction–background

Acute appendicitis is one of the most common acute surgical emergencies in children [1–3]. Historically, treatment has typically involved appendicectomy [4–7]. In the UK almost fourteen thousand appendicectomy procedures are performed every year on children under 18 years of age [8]. These procedures at the time of this study in 2017, costed on average between £2,415 and £5,055 and accounted for an annual total cost of over £60 million to the UK National Health System (NHS) [8]. In recent years the concept of treating appendicitis non-operatively thereby avoiding appendicectomy, has gained interest and some attractiveness. This treatment modality is currently being formally evaluated as an alternative to invasive surgery [9–11]. In addition to understanding the implications of these different treatment pathways on clinical and patient-centred outcomes it is important to consider the cost implications of these different treatment approaches [12]. 

We have recently completed a feasibility randomised controlled trial (RCT) comparing non-operative treatment with appendicectomy in children with uncomplicated appendicitis. The CONservative TReatment of Appendicitis in Children—randomised controlled Trial (CONTRACT) feasibility study was conducted to explore whether it is feasible and acceptable to conduct a multicentre RCT testing the effectiveness and cost-effectiveness of a non-operative treatment pathway for the treatment of acute uncomplicated appendicitis compared to appendicectomy in children. The rationale for and main results of this feasibility RCT have been reported elsewhere [9, 11]. Alongside the feasibility RCT we undertook health economic feasibility work to inform the design of a health economic analysis alongside a future clinical effectiveness trial. The full protocol for this health economic feasibility work has been published previously [13]. Here we report the findings of this health economic investigation and discuss the implications of our findings for our future work.

The value of feasibility and pilot studies in terms of avoiding weaknesses in the design, conduct, and analysis of research has been highlighted for some time now [14]. However, despite developments in this area, the value of including health economics at this early stage has been overlooked [15]. Following the relevant recommendations [15] and incorporating economic evidence into the CONTRACT feasibility study we aimed to assess data collection tools and methods and to determine indicative costs and benefits prior to progressing to a definitive RCT. The objectives of the economic sub-study were to define and refine methods of data collection and analysis and to assess two alternative HRQoL (Health Related Quality of Life) measures used to estimate QALYs (Quality Adjusted Life Years).

In terms of resource use and costs, reliable cost data forms the foundation of economic evaluations. Historically there are two methods used, bottom-up micro-costing and top-down aggregate costing [16, 17]. In this study we explored both methods and we also used PLICS data comparing our results. Patient-Level Information and Costing Systems (PLICS) [18] was initially introduced in mid-2000s but the adoption within the wider secondary care has been relatively slow. NHS England and NHS Improvement’s Costing Transformation Programme (CTP) was set up to implement PLICS across acute, mental health, ambulance and community providers and the use of PLICS methodology is set to increase in the future. The principle of reference costs for PLICS involves establishing and improving data quality and costing information within an organisation [19]. However, this also provides a valuable costing tool for health research.

Preference-based QoL instruments are used to estimate QALYs. Generic instruments used in this respect are sometime insensitive to some aspects of certain conditions [15, 20, 21]. This becomes even more challenging when the population of interest are children where there are no specific recommendations [22, 23]. However, in addition to an appropriate instrument to generate utility values, equally important is the timeframe of the analysis conducted and the intervals during which data is collected. QALYs provide a means to combine time and health preferences into a single index [24, 25]. These different elements constitute the QALY measure. Within this context we estimate the change in utility values induced by the treatment multiplied by the duration of the treatment effect to estimate the number of QALYs gained. Therefore, we also explored the implications of elements of the QALY measure such as timing of data collection and duration of a cost-utility analysis within a RCT.

Time is an important aspect of any economic evaluation, as the timing and duration of clinical events all have implications for estimating costs and benefits assessing an intervention [26, 27]. Incorporating economic evidence into an early stage of the study, the research questions we aimed to address were: (i) what are the resource use and costs of treating childhood appendicitis non-operatively as compared to appendicectomy and how do the costs of both treatment options compare to widely used NHS Reference Costs; (ii) what could be the implications of differing costing methods and data collection tools; (iii) how do two widely used preference-based QoL instruments compare and (iv) could the timing of data collection and length of the analysis affect QALYs and hence the results from a CUA.

## Methods

The full methods of the CONTRACT feasibility RCT are reported elsewhere [9, 11, 28] but briefly, children (age 4–16 years) with a clinical and/or radiological diagnosis of acute uncomplicated appendicitis were enrolled and randomly assigned to either a non-operative treatment pathway or appendicectomy. The study took place at three specialist children’s surgery centres in England and recruitment took place over a 12-month period from March 2017 until February 2018. Overall, 57 children with acute uncomplicated appendicitis were recruited to the study and followed-up to six months following randomisation.

The alongside economic analysis was carried out from the perspective of the UK health system (NHS) which underpinned the classifications and domains used in the analysis (micro-costing). It was conceptually divided into two parts: Part I, Resource Use and Costs: the development of tools and assessment of tools and methods measuring resource utilisation and costs and Part II, HRQoL: measuring QoL using two different instruments, (CHU-9D and EQ-5D-5L) used in paediatric research and assessing the impact of different instruments, and the impact of timing and duration of data collection, on utility values and QALYs.

### Part I—resource use and costing methods

**Fig. 1:**
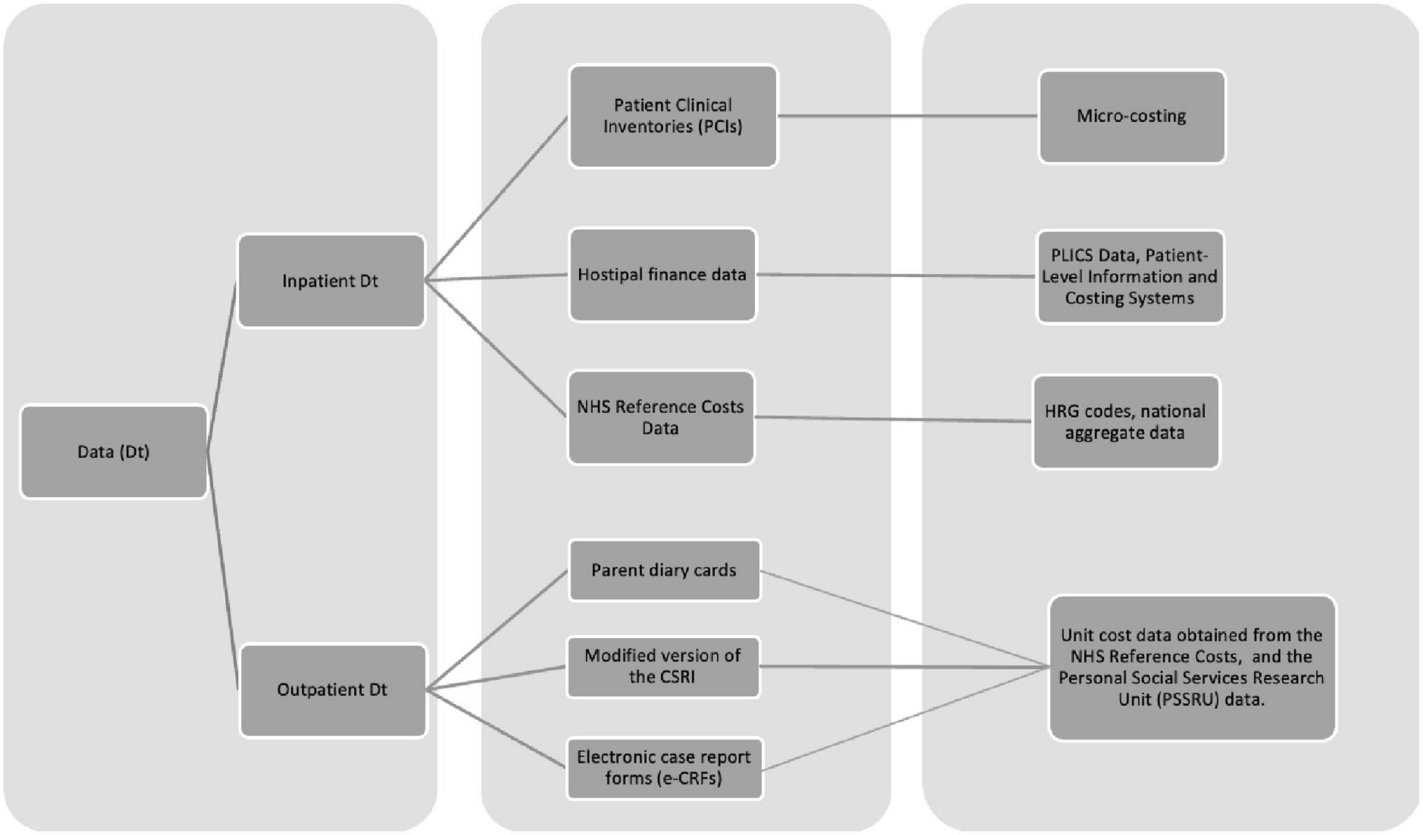
Resource use data

**Fig. 2:**
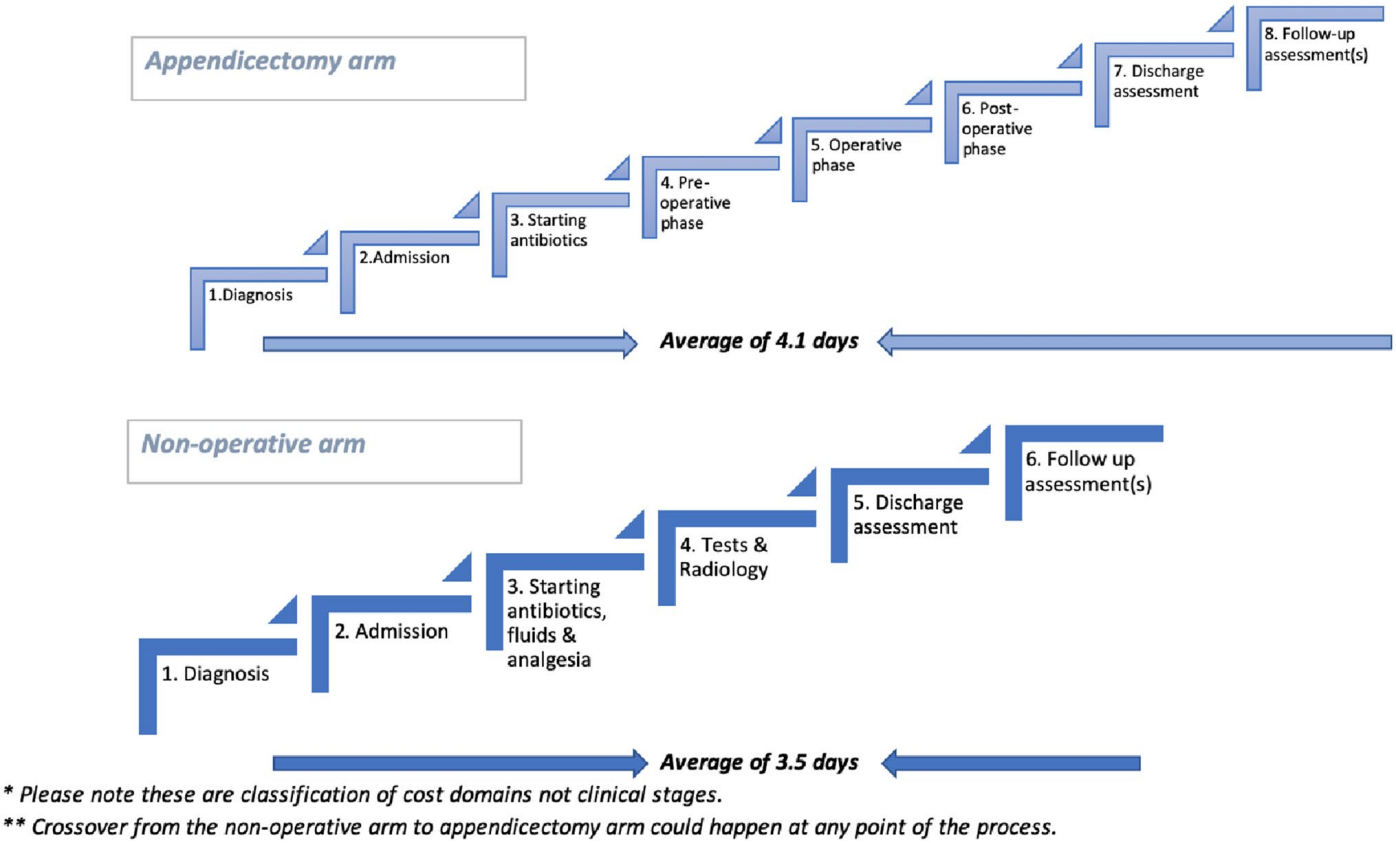
Treatment pathways for each treatment arm separated into domains

The economic implications of each stage of both trial treatment pathways were documented and the health care resource use was measured for each participant using clinical records, case report forms completed by research nurses, and Client Service Receipt Inventory (CSRI) questionnaires [29] completed by parents/carers. In our empirical investigation we sought to estimate and assess the level of agreement between data sources, the quality of the data, and the level of precision. We adopted a comprehensive approach of collecting data (Fig. 1) during the in-patient phase of treatment from hospitals, and from the wider health care system following hospital discharge (outpatient phase). To allow a full understanding of each treatment pathway and the costs associated with each component part we mapped each treatment pathway into its constituent parts (Fig. 2).

### Inpatient phase

#### Micro-costing (bottom-up costing)

Resource use data was recorded on Patient Clinical Inventories (PCIs) designed to capture the full breadth of resource use during hospitalisation and informed from hospital records including in-hospital and outpatient clinical records, laboratory and pharmacy records, diaries, radiology department records, and relevant correspondence. We first designed PCIs in collaboration with the clinical teams at each participating site. This involved identifying and mapping processes involved in service delivery for each stage of the treatment pathway, identifying and recording the relevant resource use items. Following an empirical design, the PCIs were piloted for the first 10 patients randomised in the study across all sites. This allowed us to modify PCIs based on real patient data to ensure that all aspects of variation in care pathways were captured. The finalised PCIs were completed for all patients recruited during the second six months of the study across all three participating sites. Unit cost data used in the valuation of resource utilisation was obtained from the finance department of participating hospitals. Unit cost data for resource use following initial hospital admission (e.g. outpatient and emergency appointments) was obtained from the NHS Reference Costs [30], and the Personal Social Services Research Unit (PSSRU) data [31].

#### Patient-level information and costing systems (PLICS)

During the study we learnt that one of the participating hospitals was routinely using PLICS methodology [32] to assign cost to each hospital admission. PLICS is a relatively novel approach in the UK that brings together healthcare activity with financial information and provides detailed data about how resources are used at patient-level, for example, staff, drugs, and diagnostic tests. Therefore, in addition to comparing our micro-costing derived cost to NHS Reference Cost (macro-costing), we also included a comparison to the actual cost of treatment provided by the participating hospital using PLICS methodology. This was especially relevant for the non-operative treatment arm since non-operative treatment of appendicitis is a relatively new proposition and we needed to define the treatment pathway in terms of overall resource use and costs.

#### NHS reference costs

Regarding resource use and costs during the inpatient phase, the most commonly used approach in the UK for calculating the cost of hospitalisation in an economic evaluation is to use the NHS Reference Costs (National Schedule of Reference Costs) associated with the relevant Healthcare Resource Group (HRG) code (top-down/macro-costing). This macro-costing approach reports the average costs across different hospitals for a specific HRG code. Given the uncertainty of the accuracy of the NHS Reference Costs [33] and in particular the lack of an appropriate NHS Reference Cost for the non-operative treatment pathway at the time, we used a micro-costing approach and aimed to compare cost per case with NHS Reference Cost.

### Outpatient phase

Data following discharge was collected using:

(i) Electronic case report forms (e-CRFs) which were completed by research nurses during interviews with parents/carers following discharge at 6 weeks, three months and six months post randomisation.

(ii) Parent completed diary cards which were used to record family activity as well as resource use during the 14 days immediately after discharge from hospital (e.g. oral antibiotics, pain and analgesia medications).

(iii) a modified version of the CSRI questionnaire (used to collect data on health care appointments and additional family borne costs) completed by parents/carers at 6-weeks, 3- and 6-months.

For this phase, data collected using e-CRFs, parent diary cards and the CSRI questionnaire [34] were also costed using unit cost data from the NHS Reference Costs [30], and the Personal Social Services Research Unit (PSSRU) data [31]. For the purposes of assessing data reporting tools, we assessed completeness and quality of data collected in e-CRF’s by research nurses with data reported in patient/parent completed questionnaires (diaries and CSRI).

In terms of analysis of cost data, the process we followed intended not only to identify the data required in a future RCT but also to assess quality of data in terms of missing values and accuracy. During the feasibility stage it is not appropriate to directly compare the trial treatment arms, therefore we report descriptive statistics. However, we did compare inpatient costs from the three different sources to determine the most accurate source for our future RCT. All costs are presented in 2017/18 prices, where necessary the unit costs were adjusted for inflation using the hospital and community health services (HCHS) index [31].

### Part II—preference-based HRQoL, utility values and QALYs

We used the EQ-5D-5L [35] version of the EuroQoL questionnaire which comprises 5 questions/dimensions and 5-levels of response and the CHU-9D [36–41] paediatric questionnaire which also uses 5-levels of response to 9 questions/dimensions. Using two questionnaires with the same level of responses allows comparison by providing similar level of sensitivity to marginal variations in terms of ceiling effect. The youth version of the EQ-5D-5L was not available at the time of this study, the EQ-5D-Y-5L developed in 2019 [42] is still in beta phase [43] and not officially adopted by EuroQoL. The CHU-9D is the only questionnaire that has been developed with children (7–17y) and the value set attached was obtained from a UK based general population sample. We collected both instruments from parents/carers of trial participants, responding as a proxy for their child, at baseline, discharge, 2-weeks and 6-weeks to determine any short-term difference in HRQoL that may not be apparent in later follow-up, and then at 3-months, and 6-months follow-up.

Responses from the two questionnaires were used to estimate utility values and QALYs and assess the short- and longer-term implications of the two treatments on HRQoL. Comparing the two instruments and using the full sample from both treatment groups we used the paired t-test and the non-parametric alternative the Wilcoxon signed-rank test to compare paired data. Our analysis was based on our aim of assessing which instrument shows superior performance in terms of sensitivity to change and quality of data on this patient-group, but also to assess how the different data collection points (timing) and duration of the analysis could affect the QALYs and the CUA. It is important to consider when in the process patients have returned to normal health, implying that any treatment effect is minimised. Extending the timeframe beyond the treatment effect could dilute the results of a CUA. Descriptive statistics were used to assess utility values and QALYs derived from the two instruments. The feasibility and acceptability of the CHU-9D and the EQ-5D-5L were assessed by missing values and ceiling effects.

## Results

In total 57 participants were enrolled (RCT), with 29 allocated to the non-operative treatment arm and 28 to appendicectomy. Data for all participants, with the exception of those who withdrew consent for data collection, were available for the economic analysis. Micro-costing was performed on 28 of the 30 patients enrolled in the second 6 months of the trial recruitment period (2 cases withdrew consent for ongoing data collection and were therefore excluded). These were 13 in the non-operative treatment arm and 15 in the appendicectomy arm. Baseline characteristics of participants in each trial arm were similar (full details reported elsewhere [9]*).*

### Part I—resource use and costing methods

#### Inpatient phase

Although we had originally intended to obtain unit cost data for each and every resource use item within the PCI from all three participating hospitals, this only proved possible for one participating hospital. We therefore applied the unit costs from the single hospital in the micro-costing analysis for all 28 participants regardless of treatment site. Similarly, we were only able to obtain the actual cost of treatment (PLICS data) from one participating hospital.

In-patient costs for both treatment arms are shown in Tables 1 and 2 (based on the micro-costing method, PCI data). For participants randomised to the non-operative treatment arm but undergoing appendicectomy either during the initial hospital admission or during follow-up period (readmission) all costs were included. The mean (s.d.) total cost estimates for the non-operative arm and the appendicectomy arm were £2,190 (1,332) and £4,411 (1,271) respectively. For the non-operative treatment arm, ward stay was the most significant cost driver (66%). The cost of the operation phase for patients undergoing an operation (treatment crossover) was the second largest cost driver (22%). Within the appendicectomy arm, the cost of the operation phase was the most significant cost driver (53%), with equipment and facilities costs constituting the main part (41%). This was followed by ward stay (38%). Overall, these exploratory results show the non-operative treatment arm incurred lower inpatient costs for the 6 months period compared to the appendicectomy arm.

**Table 1:** Costs (£) of the Non-Operative arm, Source: Hospital Records (PCI)

Classification	Domains	*N*	Mean	Std. Deviation
Antibiotics (ABx) phase	Antibiotics	See below in-patient medications in detail		
	Other Medications	13	10.44	(14.43)
	Consumables & Disposables	*13*	*23.91*	*(23.54)*
	Tests & diagnostics	13	3.90	(8.74)
	Other Teams	13	2.64	(9.51)
	Cost of ABx phase	*13*	*40.89*	*(44.49)*
In case of treatment failure treated with appendicectomy				
In case of treatment failure treated with appendicectomy	Pre-Op Medications	13	0.16	(0.56)
	Op Medications	13	18.70	(47.19)
	Consumables & Disposables	13	19.32	(47.32)
	Clinical Staff	13	89.49	(239.50)
	Equipment & Facilities	13	334.38	(860.40)
	Laboratory Tests	13	14.00	(34.22)
	Cost of Operation phase	*13*	*475.89*	*(1224.79)*
	POC† Medications	13	1.28	(3.60)
	POC† Consumables & Disposables	13	0.29	(0.75)
	Cost of POC† phase	*13*	*1.58*	*(4.14)*
Total in-patient Stay	Antibiotics	*13*	*93.53*	*(223.11)*
	Analgesics	*13*	*51.81*	*(60.02)*
	Ward stay (days)	13	3.54	(1.85)
	Ward stay cost	*13*	*1,450.77*	*(759.98)*
Discharge Assessment (DA) phase	Medications	13	2.49	(7.76)
	Consumables & Disposables	13	0.14	(0.46)
	Clinical Review	13	2.45	(8.83)
	Clinical Staff	13	49.45	(15.93)
	Laboratory Tests	13	8.42	(29.92)
	Cost of DA phase	*13*	*62.95*	*(32.21)*
Outpatient ABx		*13*	*1.83*	*(2.77)*
Follow-up Appointments (FuA) phase		*13*	*11.15*	*(40.19)*
Total Cost of No-Operative arm		*13*	*2,190.39*	*(1332.30)*

^†^Post-Operative & Complications (POC) phase

^*^Values in italics are subtotals for each phase which are then summed to give the total cost

^**^Note that 7 patients of the 13 underwent appendicectomy following non-operative treatment failure. Cost of appendicectomy phase for these 7 are distributed across all 13 patients who were allocated to non-operative treatment

**Table 2:** Costs (£) of the Appendicectomy arm, Source: Hospital Records (PCI)

Classification	Domains	*N*	Mean	Std. Deviation
Antibiotics (ABx) phase	Medications	See below in-patient medications in detail		
	Consumables & Disposables	*15*	*44.39*	*(34.35)*
Pre-Operative (Pre-Op) phase	Medications	15	2.29	(1.97)
	Consumables & Disposables	15	2.89	(2.89)
	Cost of Pre-Op phase	*15*	*5.18*	*(3.51)*
Operation phase	Medications	15	102.89	(17.02)
	Consumables & Disposables	15	52.43	(26.91)
	Clinical Staff	15	348.82	(209.46)
	Equipment & Facilities	15	1,827.91	(577.13)
	Laboratory Tests	15	21.85	(6.78)
	Cost of Operation phase	*15*	*2,353.90*	*(714.16)*
Post-Operative & Complications (POC†) phase	Medications	15	14.80	(18.58)
	Consumables & Disposables	15	14.81	(12.26)
	Laboratory Tests	15	1.33	(2.88)
	Radiology	15	3.87	(10.20)
	Other Teams	15	11.74	(21.80)
	Cost of POC† phase	*15*	*46.55*	*(43.20)*
*Total in-patient Stay*	Antibiotics	*15*	*53.23*	*(72.29)*
	Analgesics	*15*	*112.86*	*(182.28)*
	Ward stay (days)	15	4.13	(1.92)
	Ward stay cost	*15*	*1,694.67*	*(788.14)*
Discharge Assessment (DA) phase	Medications	15	0.41	(0.86)
	Consumables & Disposables	15	0.20	(0.73)
	Clinical Review	15	15.33	(36.11)
	Clinical Staff	15	57.42	(3.96)
	Laboratory Tests	15	0.68	(2.64)
	Cost of DA phase	*15*	*74.06*	*(39.95)*
Outpatient ABx		*5*	*0.90*	*(2.29)*
Follow-up Appointments (FuA) phase		*15*	*25.46*	*(52.72)*
Total Cost of Appendicectomy arm		*15*	*4,411.20*	*(1270.60)*

^*^Values in italics are subtotals for each phase which are then summed to give the total cost

^†^Post-Operative & Complications (POC) phase

Total costs for each treatment arm obtained by the three different costing methods are shown in Table 3. For the non-operative arm, micro-costing yields a mean cost of £2,190. PLICS methodology (provided as total per patient cost) yields a mean cost of £2,597, while the unit cost from the NHS Reference Costs varies from £553–£1,918. Of note is that at the time of this study NHS Reference Cost data for non-operative treatment was not available; therefore, a range of unit costs related to other gastrointestinal disorders were typically used. For the appendicectomy arm, the mean cost from micro-costing was £4,411, from PLICS methodology was £4,957 and NHS Reference costs data ranged from £2,415 to £5,055 (average £3,515) dependent on different severity levels.

**Table 3:** Comparison of Costing Methods: (i) Micro-costing, (ii) PLICS data-cost of treatment as provided by hospital finance department and (iii) NHS Reference costs (HRG codes)

Method of Costing	Mean (s.d.)
(i) Total Costs as per micro-costing (*n* = 28)	
No-Operative arm	2,190 (1332)
Appendicectomy arm	4,411 (1271)
(ii) PLICS data (*n* = 14)	
Non-Operative arm	2,597 (655)
Appendicectomy arm	4,957 (2431)
(iii) NHS Reference costs, 2017/18	
	Range of Unit Costs
Paediatric Other to Major Gastrointestinal Disorders*	553–1,918
Appendicectomy Procedures, 18 years and under**	2,415–5,055

^*^No HRG code available for non-Operative treatment at the time. HRG codes used: (Paediatric Other Gi disorders Score 0 to Paediatric Major Gi Score 3–4) PF26C, PF25E, PF25C –

^**^HRG codes: (Appendicectomy Procedures, < 18y, CC Score 0–3 +) FZ20M, FZ20L, FZ20K

#### Outpatient phase

Overall, the non-operative treatment arm incurred higher outpatient resource use and costs £67.54 (94.66) for the 6 months period compared to the appendicectomy arm £39.20 (75.84). Data for each component of the outpatient phase is shown in Tables (OR) A and B (supplementary material). We noted differential completion rates between sources of data with higher completion rates from e-CRFs (data recorded during interview by research nurses) than from CSRI questionnaires (completed by parents/carers). The CSRI completion rate varied from 14 to 46% across treatment arms and timepoints of data collection, making the reporting extremely poor for the whole study period such that overall, only 3 patients completed the CSRI questionnaire at all time points. Conversely complete e-CRF data was obtained for 38 participants allowing us to estimate costs for the full duration of the study. Reporting from CSRI was only meaningful at 6-weeks [9]. The same applies to the quality of data where missing values within the completed questionnaires were more apparent in the CSRI data. Despite this, both data collection methods revealed that primary care outpatient resource use was limited in both arms, indicating that the majority of healthcare costs are incurred in secondary care settings (inpatient phase).

In terms of family borne costs (out of pocket expenses) due to their child’s hospitalisation, the majority of parents only reported travel and parking costs. Table (OR) C presents family-borne costs as reported at 6-weeks following discharge. The table also shows days lost from school and days lost from work for parents/carers during the 6-week period following discharge from hospital (relatively small sample).

### Part II—preference-based HRQoL, utility values and QALYs

Health utility values from both HRQoL instruments at all timepoints are shown in Table 4. Overall completion rates for the EQ-5D-5L were higher at all timepoints than for the CHU-9D. Complete data (i.e. completed both instruments across all timepoints) was only available for 28 participants (13 in non-operative treatment arm and 15 in appendicectomy arm) for EQ-5D-5L and for 18 (8 and 10 respectively) for CHU-9D, and thus QALYs could be calculated for these only (Table 5). The 2-week timepoint had the lowest completion rate for both instruments; we note that this was the only timepoint at which instrument completion was requested without research nurse support. Both instruments appeared sensitive to change over time and utility values at discharge were higher for the non-operative arm compared to the appendicectomy arm. The difference between treatment arms at hospital discharge was apparent for all dimensions assessed (mobility, self-care, usual activity, pain, anxiety, and depression; data not presented). The difference between the two arms was smaller at 2-weeks and utility values had normalised by 6-weeks, remaining normal (full health) at later timepoints.

**Table 4:** Health related quality of life (HRQoL) utility values

Timing of assessment	Study arm	EQ-5D-5L			CHU-9D		
		*N*	Mean	Standard Deviation	*N*	Mean	Standard Deviation
Baseline	Non-operative arm	28	0.532	(0.34)	25	0.605	(0.18)
	Appendicectomy arm	28	0.564	(0.33)	23	0.571	(0.13)
Discharge	Non-operative arm	26	0.920	(0.10)	21	0.895	(0.08)
	Appendicectomy arm	27	0.721	(0.26)	22	0.687	(0.14)
2-weeks	Non-operative arm	13	0.988	(0.03)	9	0.972	(0.05)
	Appendicectomy arm	15	0.894	(0.31)	12	0.862	(0.17)
6-weeks	Non-operative arm	27	0.962	(0.07)	24	0.945	(0.06)
	Appendicectomy arm	26	0.976	(0.05)	23	0.970	(0.04)
3-months	Non-operative arm	27	0.976	(0.05)	20	0.949	(0.09)
	Appendicectomy arm	27	0.993	(0.02)	23	0.974	(0.04)
6-months	Non-operative arm	25	0.995	(0.02)	20	0.974	(0.05)
	Appendicectomy arm	27	0.984	(0.06)	23	0.967	(0.09)

**Table 5:** Quality Adjusted Life Years (QALYs) as determined for each QoL instrument

	Study arm	EQ-5D-5L			CHU-9D		
		*N*	Mean	Standard Deviation	*N*	Mean	Standard Deviation
QALYs Baseline to 6-months	Non-operative arm	13	0.973	(0.03)	8	0.943	(0.04)
	Appendicectomy arm	15	0.965	(0.04)	10	0.962	(0.02)

In terms of health utility values and QALYs (Table 4 and 5 respectively) the two instruments produced marginally different values across all timepoints for both treatment arms. Comparing utility values and QALYs from the two instruments, we used the full sample from both treatment arms (paired statistical test). Utility values produced at discharge, 2w, and 6w showed no statistically significant differences between the two instruments (p-value p > 0.05). However, assessing the 3 m and 6 m results, the data from EQ-5D-5L was right-skewed indicating a ceiling effect, and there were statistically significant differences between the two instruments (p-value p < 0.05). QALYs at 6w and 3 m were not statistically significantly different between the two instruments, whereas at 6 m QALYs were statistically significantly different. This resulted to higher utility values from EQ-5D-5L at 6 m than from CHU-9D. The ceiling effect observed in our results, when patients returned to full health, was more prevalent in EQ-5D-5L even at 3 m: at 3 m 83% of patients were classified at full health according to the EQ-5D-5L, compared to 53% according to the CHU-9D. At 6 m this was 90% and 65% respectively.

## Discussion

In this economic sub-study, we assessed alternative data collection tools and methods for both resource use and HRQoL. We defined treatment pathways in health economic terms (Fig. 2) to ensure that all relevant costs were included in our micro-costing and to help identify the most significant cost drivers to consider in future research. From this we can propose a framework for a cost-effectiveness and cost-utility analysis in our future work and for the first time provide an estimate of treatment costs for the non-operative treatment arm.

Our comparison of different methods for estimating in-hospital treatment costs demonstrated close alignment between micro-costing results and PLICS data provided by hospital finance. Although PLICS data was only available for one hospital during our study period there is increasing uptake of the PLICS methodology nationally in the UK such that we anticipate it being more readily available in the future [18]. In 2011 less than half of NHS trusts had patient-level information and costing systems (PLICS) in place. Yet when implemented, PLICS provides a vast array of useful and accurate data on spending per case against income that cannot be provided by traditional costing methods [44]. PLICS data is less time intensive and equally accurate as the micro-costing approach. We expect that PLICS data will be more widely available in the future and will play a significant role in future health economics research. Whilst the micro-costing approach was feasible it was time consuming and unlikely to be sustainable in a larger trial. Given the development of PLICS methodology, it has the potential to be an extremely reliable data source for use in health economic clinical research. There was less agreement in costs obtained using individual-patient data, either by micro-costing or PLICS, compared to the aggregate NHS Reference Costs data (macro-costing). Within the NHS Reference Cost there is a wide range of HRG codes that could be applied (Table 3) but we would anticipate most children with uncomplicated appendicitis to be assigned to the least costly of these. Thus, overall, the NHS Reference Cost (macro-costing) likely underestimates the true costs of treatment for both treatment arms. On balance we believe using PLICS methodology will be the most efficient and accurate method to define costs per participant in our future RCT.

Our results demonstrate that the ward stay, and the operation cost are the two main cost drivers. However, we note differences in other cost items between treatment arms that would be overlooked were a future trial to only consider these key cost drivers alone, not least the cost implications of needing surgery in the non-operative treatment pathway, where ward stay alone would be the anticipated single cost driver. Whilst it may therefore be tempting to base a future economic analysis on key cost drivers alone, we believe that a full detailed analysis incorporating all hospital related costs is preferable and more precise. The more widespread availability of PLICS data will greatly facilitate recording these costs in full.

Assessing the tools used for data collection, our study shows that superior quality of data was collected by research nurses during interviews with parents/carers (e-CRF) as compared to parent-completed questionnaires (CSRI). The importance of research nurse involvement in health economic data collection is also supported by higher completion rates of HRQoL instruments when done with research nurse support. This knowledge will inform our future work and is likely transferable across different studies.

In terms of HRQoL instruments, our assessment indicates that both instruments provided similar results overall, yet the instruments produce different utility values and consequently QALYs, indicating that different HRQoL measures may not be interchangeable. This has been highlighted elsewhere [45–48]. In terms of data completeness, EQ-5D-5L was superior (fewer missing values) indicating that perhaps shorter instruments may be more acceptable.

It is well documented that EQ-5D-3L and EQ-5D-Y which is also at 3-level, produces a ceiling effect (CE). The CE is defined as the proportion of responders reporting “no problems”. The greater the CE, the less sensitive the instrument. In an attempt to minimise the CE we used the EQ-5D-5L version. The results show that the 5L version, despite minimising perhaps the problem, still presents a valid reason for concern. Our results were consistent with similar findings [49]. Therefore, given that CHU-9D is developed for children and has smaller CE, the observed differences might not be significant enough to justify switching to an instrument that uses adult-based valuation. The issue is to a great extent a matter of judgement.

An important observation is the change in utility values over time, and in particular in the short term (at discharge and 2 weeks). There is a suggestion that utility values have normalised by 6 weeks, which highlights the need to collect short-term data and to carefully consider the duration for which QALYs will be estimated to assess cost-effectiveness. Failure to collect short-term data in a future trial could result in missing differences in HRQoL between treatment arms, and indeed further repeated measures between discharge and six weeks may enable any such differences to be identified. Equally important is the length of the assessment considering that long duration might dilute the results and could have significant effect in assessing QALYs and cost-effectiveness. There may be no added benefit in collecting QoL data beyond 6 weeks since we would not (based on our data) anticipate any further change over time. However, failure to record utility values over the entire follow-up duration of a trial may result in failure to detect important changes that we have not detected here based on small numbers and which may be associated with related clinical events, such as recurrent appendicitis or late complications. Whilst reducing the number of timepoints at which HRQoL is recorded may reduce burden to participants and research cost, and result in fewer missing data points, focussing resources on the most important timepoints and supporting parents in completing the questionnaires is likely to provide more reliable data. In terms of a future cost-utility analysis assessing both short- and longer-term cost per QALY seems the most balanced approach.

## Strength and weaknesses

We consider a specific strength of this study to be the detailed identification and quantification of costs incurred in each treatment pathway in our micro-costing process. Our ability to measure these costs was especially important due to the lack of unit cost data (HRG code) for the non-operative treatment pathway for acute appendicitis. A potential weakness of the study is that the data informing our micro-costing analysis used unit costs from a single participating hospital. There are known to be differences in the cost of providing similar services between different NHS trusts resulting from differences in contractual arrangements and estates costs amongst others. Therefore, the micro-costing results may not be representative nationally for all hospitals. Additionally, the PLICS data was only available from one hospital. However, our results strongly suggest that the two main cost drivers identified in this study are likely to be the same for other hospitals, given how dominant they were in the overall cost estimates for each treatment pathway. Overall, we achieved our aim to provide a detailed assessment of data collection tools and methods, and identified important aspects that should be taken into account in our future RCT.

## Conclusions

We have generated a first indication of costs for treatment of uncomplicated appendicitis in children using operative and non-operative treatment pathways and identified important considerations for costing and measuring HRQoL to inform our future RCT. Given the small number of participants and potential between-patient heterogeneity we hazard against drawing firm conclusions from these data. Our data for the non-operative treatment pathway may be useful in informing national tariffs for non-operative treatment given the paucity of data that currently exists.

## Supplementary Information

Below is the link to the electronic supplementary material.

Supplementary file1 (DOCX 29 KB)

## Data Availability

Data requests should be submitted to the corresponding author for consideration.
