# Supplementary material for: Assessing micro- vs macro-costing approaches for treating appendicitis in children with appendicectomy or non-operatively
Source: Qual Life Res. 2023 Jun 7;32(10):2987–99. doi: 10.1007/s11136-023-03442-w (PMC10473981; doi:10.1007/s11136-023-03442-w)
Supplement: Supplementary file 1 — Supplementary file1 (DOCX 29 KB) [file 11136_2023_3442_MOESM1_ESM.docx]

**Assessing micro- vs macro-costing approaches for treating appendicitis in children with appendicectomy or non-operatively**

Maria Chorozoglou^1,*^, Isabel Reading ^2^, Simon Eaton ^3^, Naqvi, Shehryer^4^, Caroline Pardy^4^, Keren Sloan^5^, Christina Major^5^, Natasha Demellweek^6^, and Nigel J Hall ^1,5^,

^1^ Faculty of Medicine, University of Southampton, Southampton, UK
^2^ School of Primary Care, Population Sciences and Medical Education, Faculty of Medicine, University of Southampton, Southampton, UK
^3^ University College London Great Ormond Street Institute of Child Health, Department of Population Health Sciences, University College London, London, UK
^4^ St George’s University Hospital NHS Foundation Trust, London, UK

^5^ Southampton Children’s Hospital, Southampton, UK

^6^ Alder Hey Children’s Hospital, Liverpool, UK

^*^ Corresponding author Email:  [M.Chorozoglou@soton.ac.uk](mailto:M.Chorozoglou@soton.ac.uk)

# Online Resource

Table (OR) A, Cost (£) related to resource use during 6-month follow-up period (source e-CRF)

| ***Classification*** | ***Study arm*** | ***N*** | ***Mean*** | ***(s.d.)*** |
| --- | --- | --- | --- | --- |
| ***Costs: baseline to 6 months*** | | |  |  |
| A&E visits | Non-operative arm | 18 | 41.53 | (85.89) |
|  | Appendicectomy arm | 20 | 14.95 | (46.02) |
| GP visits | Non-operative arm | 18 | 11.89 | (19.73) |
|  | Appendicectomy arm | 20 | 10.70 | (19.01) |
| Practice nurse | Non-operative arm | 18 | 0.75 | (3.18) |
|  | Appendicectomy arm | 20 | 0.68 | (3.02) |
| Hospital Outpatient | Non-operative arm | 18 | 5.71 | (17.63) |
|  | Appendicectomy arm | 20 | 11.99 | (27.85) |
| Laboratory tests | Non-operative arm | 18 | 0.79 | (1.62) |
|  | Appendicectomy arm | 20 | 0.88 | (2.21) |
| Walk in centre & other health related care | Non-operative arm | 18 | 7.57 | (17.41) |
|  | Appendicectomy arm | 20 | - | - |
| ***Total Costs: baseline to 6 months*** | | |  |  |
| ***Summation of all services*** | ***Non-operative arm*** | ***18*** | ***67.54*** | (94.66) |
|  | ***Appendicectomy arm*** | ***20*** | ***39.20*** | (75.84) |

Table (OR) A presents the mean (s.d.) cost values (e-CRF) for the trial duration (baseline to 6-months) for each cost category.

Table (OR) B, Cost (£) of resources use, all time points (source e-CRF)

| ***Classification*** | ***Study arm*** | ***N*** | ***Mean*** | ***(s.d.)*** |
| --- | --- | --- | --- | --- |
| ***Costs baseline to 6 weeks*** | | | | |
| A&E visits | Non-operative arm | 23 | 13.00 | (62.30) |
|  | Appendicectomy arm | 23 | 13.00 | (43.10) |
| GP visits | Non-operative arm | 23 | 5.58 | (14.70) |
|  | Appendicectomy arm | 23 | 16.75 | (38.10) |
| Practice nurse | Non-operative arm | 23 | 0.59 | (2.80) |
|  | Appendicectomy arm | 23 | 0.59 | (2.80) |
| Hospital Outpatient | Non-operative arm | 23 | 2.98 | (9.90) |
|  | Appendicectomy arm | 23 | 5.96 | (22.30) |
| Laboratory tests | Non-operative arm | 23 | 0.44 | (1.80) |
|  | Appendicectomy arm | 23 | 0.80 | (2.10) |
| Walk in centre & other health related care | Non-operative arm | 23 | 5.92 | (15.60) |
|  | Appendicectomy arm | 23 | - | - |
| ***Total Costs*** | ***Non-operative arm*** | ***23*** | ***28.51*** | ***(66.00)*** |
|  | ***Appendicectomy arm*** | ***23*** | ***37.10*** | ***(83.00)*** |
| ***Costs 6w to 3 months*** | | | | |
| A&E visits | Non-operative arm | 21 | 35.60 | (65.20) |
|  | Appendicectomy arm | 24 | 12.46 | (42.20) |
| GP visits | Non-operative arm | 21 | 6.11 | (15.30) |
|  | Appendicectomy arm | 24 | 1.78 | (8.70) |
| Practice nurse | Non-operative arm | 21 | - | - |
|  | Appendicectomy arm | 24 | - | - |
| Hospital Outpatient | Non-operative arm | 21 | 3.26 | (10.30) |
|  | Appendicectomy arm | 24 | 2.86 | (9.70) |
| Laboratory tests | Non-operative arm | 21 | 2.16 | (9.90) |
|  | Appendicectomy arm | 24 | - | - |
| Walk in centre & other health related care | Non-operative arm | 21 | 0.64 | (1.50) |
|  | Appendicectomy arm | 24 | 0.11 | (0.50) |
| ***Total Costs*** | ***Non-operative arm*** | ***21*** | ***47.14*** | ***(78.60)*** |
|  | ***Appendicectomy arm*** | ***24*** | ***17.10*** | ***(48.80)*** |
| ***Costs 3m to 6 months*** | | | | |
| A&E visits | Non-operative arm | 21 | - | - |
|  | Appendicectomy arm | 23 | - | - |
| GP visits | Non-operative arm | 21 | 4.08 | (12.90) |
|  | Appendicectomy arm | 23 | - | - |
| Practice nurse | Non-operative arm | 21 | - | - |
|  | Appendicectomy arm | 23 | - | - |
| Hospital Outpatient | Non-operative arm | 21 | - | - |
|  | Appendicectomy arm | 23 | 2.98 | (9.90) |
| Laboratory tests | Non-operative arm | 21 | - | - |
|  | Appendicectomy arm | 23 | - | - |
| Walk in centre & other health related care | Non-operative arm | 21 | - | - |
|  | Appendicectomy arm | 23 | - | - |
| ***Total Costs*** | ***Non-operative arm*** | ***21*** | ***4.08*** | ***(12.90)*** |
|  | ***Appendicectomy arm*** | ***23*** | ***2.98*** | ***(9.90)*** |

Table (OR) B presents costs for each category at 6w, 3m, 6m alongside total costs for each period.

Table (OR) C, Family borne Costs (£), Source: CSRI 6-weeks assessment

| ***Classification*** | ***study group*** | ***N*** | ***Mean*** | ***(s.d.)*** |
| --- | --- | --- | --- | --- |
| **Travel cost** | Non-operative arm | 16 | 5.44 | (9.29) |
|  | Appendicectomy arm | 23 | 4.87 | (10.40) |
| **Parking cost** | Non-operative arm | 16 | 3.00 | (6.41) |
|  | Appendicectomy arm | 23 | 3.74 | (9.12) |
| **Childcare cost** | Non-operative arm | 16 | - | - |
|  | Appendicectomy arm | 23 | - | - |
| **Other household costs** | Non-operative arm | 16 | - | - |
|  | Appendicectomy arm | 23 | - | - |
| **Food cost** | Non-operative arm | 0 | - | - |
|  | Appendicectomy arm | 0 | - | - |
| **Other** | Non-operative arm | 0 | - | - |
|  | Appendicectomy arm | 0 | - | - |
| **School days lost (days)** | Non-operative arm | 10 | 8.65 | (9.22) |
|  | Appendicectomy arm | 23 | 7.78 | (5.18) |
| **Employment days lost (days)** | Non-operative arm | 16 | 2.44 | (4.80) |
|  | Appendicectomy arm | 24 | 3.42 | (4.49) |
| **CSRI time taken to complete (min)** | Non-operative arm | 11 | 7.27 | (4.67) |
|  | Appendicectomy arm | 20 | 6.05 | (3.03) |

Table (OR) C presents family-borne costs as reported at 6-weeks following discharge. The table also shows days lost from school and days lost from work for parents/carers during the 6 weeks period following discharge from hospital.
